# Linking the trust of industrial entrepreneurs on elements of ecosystem with entrepreneurial success: Determining startup behavior as mediator and entrepreneurial strategy as moderator

**DOI:** 10.3389/fpsyg.2022.877561

**Published:** 2022-11-01

**Authors:** Zia Ur Rehman, Muhammad Arif, Habib Gul, Jamshed Raza

**Affiliations:** ^1^Department of Management Sciences, Bahria University, Islamabad, Pakistan; ^2^Dean Faculty of Economics, Kardan University, Kabul, Afghanistan; ^3^Khushal Khan Khattak University, Karak, Pakistan

**Keywords:** trust on experts & enterprises, trust on media, trust on government, startup behavior, entrepreneurial strategy, entrepreneurial success, industrial entrepreneurs, entrepreneurial ecosystem

## Abstract

**Purpose:**

This study aimed to apply “multi-criteria decision approach and attitude-change theory” to examine post-COVID-19 impact on entrepreneurial mindset by investigating the link between entrepreneurs social capital (trust on three elements of ecosystem i.e., experts & enterprises, media, and government) and entrepreneurial success (both individual and organizational). Specifically, this study analyzed entrepreneurs' dispositional factor (startup behavior) as an underlying mechanism to bridge trust and entrepreneurial success. Furthermore, it also analyzed entrepreneurs' situational factor (entrepreneurial strategy) as boundary condition.

**Design/methodology/approach:**

We applied time-lagged data collection from 505 industrial entrepreneurs. Survey method was used for data collection. A 7-point Likert scale was used for the respondent response. Hayes developed PROCESS models 4 and 7 were used to test the hypothesis.

**Findings:**

The direct impact of trust on three elements of the ecosystem was found significantly positive on both startup behavior and entrepreneurial success. The direct impact of startup behavior on entrepreneurial success is also significantly positive. The impact of startup behavior on indirect mediation between trust and entrepreneurial success is visibly positive. The moderated and moderated mediation impact of entrepreneurial strategy found positively significant at low and medium values. However, this study found an insignificant moderated impact at high values of entrepreneurial strategy between trust on media and startup behavior. Furthermore, this study also found insignificant moderated mediation impact at high values of entrepreneurial strategy by interacting with two elements of ecosystem (trust on media and trust on government) through startup behavior on entrepreneurial success.

**Originality/value:**

The authors suggested that startup behavior is an underlying mechanism through which industrial entrepreneurs trust achieved desired entrepreneurial success. The authors also suggested that the influencing role of “*low level of entrepreneurial strategy”* in comparison with “*high level entrepreneurial strategy”* is more helpful to achieve entrepreneurial success.

**Implications:**

This study contributed to the literature on entrepreneurial strategy for its conditional indirect moderated impact on startup behavior and moderated mediation impact on firm entrepreneurial success. It also contributed to owners of the manufacturing industry for their startup behavior as an underlying mechanism through which trust influences entrepreneurial success.

## Introduction

Post COVID-19 life on the Planet Earth has brought significant changes in every academic discipline. The entrepreneurship discipline has also been affected significantly by COVID-19 (Pramono et al., 2021). Optimistically, COVID 19 has an impact on the entrepreneurial mindset as it found new ways of entrepreneurial success (Rashid and Ratten, 2021). In the negative way, COVID-19 affects the entrepreneurial mindset by increasing the cost of doing business (Shafi et al., 2020; Sarah et al., 2021).

Entrepreneurial success, undisputedly, is a significant outcome for entrepreneurial mindset under rising global economies (Chandna, 2022). In the 21st century post-COVID-19 world, humans are witnessing a new era of entrepreneurial mindset in the form of regional integration, regional cooperation, economic corridors, and one-belt-one-road (Farooq and Khawaja, 2020). Among this regionalism, individual- and firm-level entrepreneurial success are deemed as an important contemporary issue (Doern et al., 2019; Thompson et al., 2020). In this connect, numerous scholars have suggested different contemporary issues such as success i.e., entrepreneurial development (Unger et al., 2011), successful venture, ventures growth and ventures sustainability (Johannisson, 2017), firm profits and leading in competition (Chen and Steinwender, 2021), and so on. In these previous studies, three opinions prevail for entrepreneurship: *One opinion* brings positive entrepreneurial success (a mindset to earn profits through new and innovative startups) by arguing firms entrepreneurial success like innovation and new ways of doing business for maximum customers satisfaction (Marom et al., 2019); *the second opinion* also highlighted positive side of entrepreneurial success by arguing that individual-level success as entrepreneurial success such as entrepreneurs individual performance, entrepreneurial activities, and startups (Fried and Tauer, 2015). In contrast, *third opinion* highlighted non-positive side of entrepreneurial success like mistrust of entrepreneur on ecosystem, uncertainty for environmental issues, too much digitalization, complex data handling, ineffective entrepreneurial strategy, less startup behavior, and changing prevailing business norms toward un-civilization (Wright and Zahra, 2011; Bouncken et al., 2018; Shad et al., 2021). Hence, entrepreneurial success is contributing to the society as well as affecting the society. In this regard, well-known scholars suggest that the manufacturing industry is the backbone for entrepreneurial success (Coffey and Kabadayi, 2020).

Manufacturing industry is a major kind of entrepreneurship that affects the society in several ways including the dominance of leading global industrialists in the local manufacturing industry of less developed countries (Ramay, 2016). Imported entrepreneurship refers to Developing domestic entrepreneurship and growth through imported expertise (Markusen and Rutherford, 2002). Under recent initiatives for an economic rise, Pakistan is trying to resolve the problem (i.e., less competitive due to inflated cost of manufacturing products) faced by the local manufacturing industry through imported expertise. On the one side, the imported entrepreneurship is helping Pakistan with infrastructure development. On the other side, the local manufacturing sector of the country is facing survival challenges. For country-level cost–benefit analysis, an entrepreneurial strategy regarding recent opportunities, Pakistani industrial sector, and entrepreneurial ecosystem are very essential. Therefore, the researcher selected the industrial sector in Pakistan.

### Recent opportunities for industrialization in Pakistan

Pakistan and China singed ‘economic corridor' and industrialization projects worth 64 billion US$ in April 2015. This new industrial cooperation is known as CPEC and OBOR initiative (Farooq and Khawaja, 2020). Both Pakistan and China are committed to industrial cooperation for sustainable manufacturing growth and sustainable increase in exports (Sher et al., 2019; Sultan and Saleem, 2021). To achieve these sustainable targets, Pakistan is committed to establish nine “special economic zones” (SEZ) in different “*industrial strategic regions”* of Pakistan. China has committed to develop 11 types of infrastructures in Pakistan for motorways/highways, energy, rail, seaports, airports, communication, social & cultural, educational, health, and defense (Ramay, 2016). After the development of these infrastructures, China is also committed to transfer technology to Pakistan through five different initiatives like shifting its technological-oriented organizations (manufacturing concerns) in the newly established SEZ in Pakistan, and to provide technical/ skilled labor to Pakistani firms, train Pakistani labor, and expand Pakistani manufacturing sector through cost-effective products (Farooq and Khawaja, 2020; Rehman et al., 2020). In addition to Pakistan's commitments with China, the former is also working on multifocused industrialization (Qurat-ul-Ann et al., 2015), promoting its brand image “Made-in-Pakistan,” increasing its exports of manufacturing products. For instance, globally leading SAMSUNG started its mobile manufacturing in Pakistan, and Saudi Arabia is investing 20 billion US$ for oil-refineries and other industrial initiatives. Pakistan has established “*Special Technological Zone Authority” (STZA)* to create industrial clusters, promote innovation; offered rebates to foreign entrepreneurs; established new industrial cities like Ravi Urban City to promote industrial activities and attract foreign investments besides special tax rebates for exports-oriented manufacturing concerns including but not limited to textile, sports, medicines, leather, sugar, and so on (Farooq and Khawaja, 2020; Rehman et al., 2020; Siraj and Javaid, 2021; Sultan and Saleem, 2021). All these industrial initiatives encouraged the authors to target manufacturing concern for this study.

### Entrepreneurial ecosystem in Pakistan

Pakistani entrepreneurial ecosystem consists of various kinds of manufacturing and service sectors among which the manufacturing sector contributes 12.79% of GDP (Tunio et al., 2021). The Pakistani manufacturing sector comprises of SMEs and large-scale manufacturing units for textile, sports, automobile cars, medicines for both human and animals (Shah and Syed, 2018; Rashid and Ratten, 2021), and so on. In Pakistan, the entrepreneurial success rate is between 20 and 30% as compared to 70%−80% in developed countries (Tunio, 2020a,b). The key challenges to lower entrepreneurial rates include lack of transparency, lack of trust, lower startup behavior, lower competitive capability, and ineffective entrepreneurial strategy (Wright and Zahra, 2011; Bouncken et al., 2018; Shad et al., 2021).

To overcome these issues, Pakistan is importing foreign industrialists and offering them protected and potential profitable investment schemes for manufacturing energy products, mobiles, cars, IT products, and military products in Pakistan (Ploywarin et al., 2018; Rehman et al., 2020). This imported entrepreneurship may be helpful for Pakistan for short-term gains such as reduction in the demand for foreign currency reserves, decrease in the prices of products in Pakistan, rise in exports, increase in the products with “made-in-Pakistan,” and so on (Shad et al., 2021). In the long term, this imported entrepreneurship may create serious problems for the country i.e., OBOR is predicted as a debt trap for less developed countries; local manufacturing sector of the country may face a survival challenge since imported entrepreneurship is cost-effective so-far local manufacturers are unable to compete with; and resultantly, the imported manufactures are dominating by eliminating/substituting the local Pakistani industry (Charman et al., 2012). Pakistan needs to strengthen the indigenous industrial sector through local entrepreneurs with the help of foreign investments, and this equation will lead to industrial success. To address the issue of survival of Pakistani manufacturing industry, this study observed less-literature in previous studies.

The available previous studies link entrepreneurial success with various predictors such as entrepreneurial education, entrepreneurial skills, entrepreneurial orientation, opportunity recognition, and entrepreneurial activities (Sher et al., 2019; Yeasmin and Koivurova, 2019). The literature related to social capita also justifies the importance of several types of social capital for any society (i.e., trust, education, and social linkage). These kinds of social capital witnessed important for entrepreneurial success (Jeong, 2013). The past literature sufficiently attached the significant role the trust (as social capital) plays while providing a resource for entrepreneurial enablers, but the existing body of knowledge is still incomplete in many ways as explained in ensuing paragraphs:

First, the term “*trust”* is a domain of social capital (Lewis and Weigert, 1985). For entrepreneurial advantage, Nahapiet and Ghoshal (1998) emphasized to draw a distinction between structural, cognitive, and relational social capital due to the varying nature of each kind of social capital like trust. The rationale of “*trust”* as social capital is a complex phenomenon in predicting outcomes; more importantly, due to mistrust, over-trust, and reputation-based trust scandals (e.g., Bürge, 2019; Laroche et al., 2019). Trust assumes primary importance with various new dimensions in the entrepreneurship literature. These dimensions of trust (on entrepreneurs ecosystem) - “the system of entrepreneurial stakeholders working under relationship for entrepreneurial activities” (Cohen, 2006) – have been found very important for individual- and firm-level positive outcomes (Lins et al., 2017) because trust on the ecosystem elements such as experts, suppliers, media, service providers, enterprises, and government increases the level of entrepreneurial activities. However, while searching the past literature, the authors found deficient with research on estimating entrepreneurial success under the influence of trust on different elements of ecosystem (Mujahid et al., 2019). Such studies analyzed the link between trust and elements of ecosystem through trust on media, trust on food chain, and trust on customers purchase intentions (Lobb et al., 2007): experts trust, government trust, media trust, and enterprises trust with public risk perception and national level perceived socioeconomic success (Ploywarin et al., 2018; Rehman et al., 2020). Hence, based on the missing link, this research attempted to create a direct link of trust on experts & enterprises, media, and government with startup behavior and entrepreneurial success.
Second, dispositional factors having the intrinsic motivation and internal strength are influenced for success indicators (Moin et al., 2015; Tunio, 2020a) while imparting it from situational factors having the extrinsic motivation and external strength (Collier et al., 2015). In the existing literature, less attraction has been given for the significance of entrepreneurs' dispositional factor (startup behavior) for entrepreneurial performance outcomes. The previous research also analyzed different nexus in which entrepreneurs' dispositional factors (education, skills, behavior, attitude, and intensions) contribute to a significant underlying mechanism between entrepreneurs' intentions and venture activities (Huang et al., 2009), entrepreneurial marketing and SME performance (Nazem et al., 2021), and risk aversion and investment intensions (Zhang and Cain, 2017). In addition to these underlying mechanisms, well-known researchers such as Bao et al. (2017), Han et al. (2019), and Tunio (2020a,b) suggested that entrepreneurial firms must take research-based steps to examine a combined role of social capital (trust on elements of ecosystem) and entrepreneurs dispositional factor (startup behavior) for positive influence on entrepreneurial success. Drawing from attitude-change theory (Sherif et al., 1965), the startup behavior is treated as attitude-changed behavior to influence trust for entrepreneurial success.
Finally, entrepreneurial situational factors such as distinctive competitiveness, entrepreneurial policy, strategy, and resilience have also a positive influence to predict entrepreneurs' behaviors and outcomes (Pidduck et al., 2022). Entrepreneurial strategy - “the strategic adaptation of processes as innovative solutions for new startups” (Schumpeter, 1934; Drucker, 1985) - is a prominent entrepreneurial situational factor both for entrepreneurs' dispositional factors and entrepreneurial outcomes. These include entrepreneurial attitude and intensions (Mahmood et al., 2019), entrepreneurial behavior and entrepreneurial activities (Lu and Tao, 2010), entrepreneurial orientation and new venture success (Lechler, 2001), and so on. Prominent researchers suggested to extend the role of entrepreneurial situational factors as a boundary condition to influence entrepreneurs' behavior and performance outcomes (Omotosho and Anyigba, 2019; Shepherd and Patzelt, 2021). While identifying past literature, it comes to our notice that past literature ignored the role of entrepreneurs' situational factor (entrepreneurial strategy) as a boundary condition between trust and startup behavior. Drawing upon multicriteria decision-making (Boender et al., 1989), entrepreneurs treated strategy and trust as multi-criteria to get maximum resources from both constructs. To focus on said inadequacies in literature, this study attempted to link social capital (trust on elements of ecosystem) with entrepreneurial success (both individual success and firm success) through entrepreneurs' dispositional factor (startup behavior) as an underlying mechanism and entrepreneurs' situational factor (entrepreneurial strategy) as boundary condition.

Considering abovementioned problem and gap, this research intends to resolve that social capital, entrepreneurial situational, and dispositional factors have a significant effect on entrepreneurial success in the scenario of post-COVID 19 life. This research contributes to the literature in many ways. *Firstly*, it broadens existing understanding of entrepreneurial dispositional factor (startup behavior) and entrepreneurs' situational factor (entrepreneurial strategy) in such a way that social capital i.e., trust on elements of ecosystem (trust on media, experts & enterprises, and government) provides a necessary resource for entrepreneurial success (both individual and firm success). *Secondly*, we hypothesized the links between variables by unveiling the role of entrepreneurs' dispositional factor (startup behavior) as an underlying mechanism and entrepreneurs' situational factor (entrepreneurial strategy) as boundary conditions that contribute to map the influence of social capital as an antecedent for entrepreneurial success. Thirdly, this research draws the attention of the researchers analyzing the positive and negative effects of post-COVID-19 on entrepreneurship discipline by investigating the entrepreneurial mindset for success. Finally, the owners and top executives of manufacturing firms can learn about a novel comprehensive research framework based on six factors for the implementation of startup behavior under the contingent role of entrepreneurial strategy by interacting through social capital with low and high entrepreneurial strategy.

## Literature review

### Theoretical support

#### Multicriteria decision-making approach

Multicriteria decision-making (MCDM) was proposed as a supportive approach for a pairwise assortment of multiple complex phenomena for best decisions (Boender et al., 1989). This approach encourages rational decision-making among multiple domains from multiple choices (Triantaphyllou, 2000; Roblek et al., 2021). This approach is helpful to develop the proposed hypothesis of this study, for example, trustable environment is helpful to influence startup behavior and entrepreneurial strategy as a multi-criterion for entrepreneurs' behavior, successful ventures, and country-level improvement in national economic indicators (Junaid et al., 2020; Ezennia and Mutambara, 2022).

#### Attitude-change theory (ACT)

This theory was proposed for decision-making where human attitude, behavior, and judgment influenced the decisions (Sherif et al., 1965). This theory assisted entrepreneurs to make decisions by evaluating self-dispositional factors in comparison with situational factors (Lorenz et al., 2021; Tormala and Rucker, 2022). Attitude-change theory is also supportive to develop a proposed hypothesis of this study, for example, drawing upon attitude-change viewpoint; the trust on different elements of ecosystem changes the entrepreneurs' behavior by providing appropriate change-attitude to be substituted for the achievement of success. Here, the term change-attitude as a dispositional factor illustrates behavioral-change which is like the theories (Sherif et al., 1965) that changing-attitude (startup behavior) directly or indirectly leads to achieve success.

### Entrepreneurs trust, startup behaviors, and entrepreneurial success

Considering the importance of trust for entrepreneurial startups (Kaiser and Berger, 2021), the significance of trust on different elements of the entrepreneurial ecosystem seems apparent for best performance (Ren et al., 2016). The trust is considered as social capital influencing various behaviors and performance outcomes. Individuals considered trust as: “a firm believes in reliability” (Lewis and Weigert, 1985; Fukuyama, 1995). Firms considered trust as: “stakeholders can rely on offers of firm, either written or oral” (Braithwaite and Levi, 2003).

Startup behavior is “an ability of the entrepreneurs to do some behavioral activities for new startups in different situations.” (Bărbulescu et al., 2021). Entrepreneur is “a person who started a new profit center.” Entrepreneurial success is determined as “creating number of successful new startups” (Ezennia and Mutambara, 2022). These definitions illustrate two valued aspects of entrepreneurship: “*entrepreneurial situational factors”* where an entrepreneur used external strength (trust on different elements of ecosystem i.e., trust on experts, media and government) in combination with “*dispositional factors”* where an entrepreneur used internal strength (startup behavior) holding and revealing to influence successful ventures (Lechler, 2001; Neumeyer and Santos, 2018). These two aspects of entrepreneurs differentiate successful ventures from ineffective ventures.

The trust is effective for both dispositional factors (behavior, attitudes, and beliefs) and situational factors (individual performance, firm performance, and goals achievements) (Lobb et al., 2007; Ploywarin et al., 2018). At individual level, trust influences the positive relationship of employees with the team leader (Gill, 2008), an adaption of cross-cultural practices (Costigan et al., 2006), employees' citizenship behavior (Appelbaum et al., 2004), and so on. At the firm level, trust influences entrepreneurial success (Gupta and Mirchandani, 2018), firm's overall performance (Montiel Campos, 2017), firm's growth (Qurat-ul-Ann et al., 2015), firm's sustainability (Santos, 2020), and firm innovation (Paunov, 2016).

Trustable environment is helpful to influence startup behavior and success as a multicriteria or attitude-change for entrepreneurs' behavior, successful ventures, and country-level improvement in national economic indicators (Junaid et al., 2020; Ezennia and Mutambara, 2022). Looking at the above-mentioned context, trust on elements of ecosystem may be enhanced by considering individual behavioral outcomes and firm-level performance as multicriteria for the entrepreneurs (Lado et al., 2008). Trust becomes significant for firm-level output preferably by changing the behaviors of individuals. This changing behavior based on attitude-change ultimately enhances the performance of firms (Lyubovnikova et al., 2017). Besides these studies on trust, the construct “trust on elements of ecosystem” is still required to be studied under entrepreneurship. Trust needs more understanding of three elements of ecosystem i.e., trust on experts/enterprises, media, and government (Ploywarin et al., 2018; Rehman et al., 2020). Hence, we hypothesize a relationship between trust, behavior, and success under the entrepreneurship discipline:

***H1(a):*** *Entrepreneurs' trust on experts* and *enterprises has a positive impact on entrepreneurs startup behaviors*.
***H1(b):*** *Entrepreneurs' trust on media has a positive impact on impact on entrepreneurs startup behaviors*.
***H1(c):*** *Entrepreneurs' trust on Government has a positive impact on impact on entrepreneurs' startup behaviors*.
***H2(a):*** *Entrepreneurs' trust on experts & enterprises has a positive impact on entrepreneurial success (individual success and firm success)*.
***H2(b):*** *Entrepreneurs' trust on media has a positive impact on entrepreneurial success (individual success and firm success)*.
***H2(c):*** *Entrepreneurs' trust on Government has a positive impact on entrepreneurial success (individual success and firm success)*.
***H3:*** *Entrepreneurs' startup behaviors have a positive impact on entrepreneurial success (individual success and firm success)*.

### Entrepreneurs' dispositional factor (startup behaviors as underlying mechanism or mediator)

Human behaviors are the activities of the individual or groups to behave according to dispositional and situational circumstances (Skinner, 1965). In entrepreneurship, startup behavior is an emerging construct. Startup behavior is seen as a positive mediator between startup intensions and startup attitude (Zanger and Geissler, 2018). New startups play a significant role in entrepreneurial education and national-level economic growth (Petrenko et al., 2019). Startup capital (as a multicriteria) is found to be a positive mediator for the influence of psychological capital on entrepreneurial success (Baluku et al., 2016).

Drawing from multicriteria decision-making (Boender et al., 1989), trust plays an influencing role by changing individual behaviors; individuals use trust as multicriteria for a link with firm success (Bi et al., 2021). Similarly, the trusting beliefs influenced the purchase behavior of the customers through multicriteria i.e., branding or manufacturing; resultantly, the country-level manufacturing industry gained high growth (Coffey and Kabadayi, 2020). However, besides this research on behavior, the construct “startup behavior” is still required to be studied for entrepreneurs' trust and entrepreneurial success where more understanding of the construct trust in entrepreneurship for trust on government, media and experts/enterprises, startup behaviors, and entrepreneurial success is needed to be explored (Lobb et al., 2007; Ploywarin et al., 2018). This argument may also be supported through attitude-change viewpoint.

Drawing upon attitude-change viewpoint; the trust on different elements of ecosystem changes the entrepreneurs' behavior by providing appropriate change-attitude to be substituted for the achievement of success. Here, the term change-attitude as a dispositional factor illustrates behavioral-change which is like the theories (Sherif et al., 1965) that changing-attitude (startup behavior) directly or indirectly leads to achieve success. Therefore, this study states that an increased trust on experts & enterprises, media, and government leads to behavioral change in entrepreneurs (startup behavior) resultantly achieves successful startups. Hence, keeping in view these arguments, the present research investigates the following indirect relationships between trust, behavior, and success:

***H4(a):*** *Entrepreneurs' startup behaviors have positively mediate the nexus between entrepreneurs trust on experts & enterprises and entrepreneurial success (individual success and firm success)*.
***H4(b):*** *Entrepreneurs' startup behaviors have positively mediate the nexus between entrepreneurs trust on media and entrepreneurial success (individual success and firm success)*.
***H4(c):*** *Entrepreneurs' startup behaviors have positively mediate the nexus between entrepreneurs trust on government and entrepreneurial success (individual success and firm success)*.

### Entrepreneurs' situational factor (entrepreneurial strategy as boundary condition or moderator)

Entrepreneurial strategy is a “strategic process” and is defined as “the strategic adaptation of processes as innovative solutions for new startups” (Schumpeter, 1934; Drucker, 1985). The other scholars, e.g., Covin and Slevin (1990), defined as “repeated and continual attempt to achieve competitive advantage through innovation”. Entrepreneurial Strategy (EStrg) is a form of firm policy for risk taking that uses proactiveness for change, innovation, and best utilization of resources by learning from past mistakes (Zhao et al., 2021). Hence, EStrg is considered as a situational influencing construct deemed as situational factor through which entrepreneurs enhance firm innovative performance (Feldman, 2014).

The term entrepreneurial strategy was invented in 1930s; however, in recent decades, EStrg attracted high intentions (e.g., Gans et al., 2019; Shepherd and Patzelt, 2021; Cob, 2022). From these studies, scholars examined the antecedents and consequences of entrepreneurial strategy for firms, groups, and individuals such as network ties, capital gains, customers' orientation, firm performance, employee training, cross-functional collaboration, entrepreneurial opportunity, and entrepreneurial experience. In contrast, other scholars examined it as an attribute that exerts influence as a moderator between proactive orientation and entrepreneurial performance (Gao et al., 2018); contingent environment and agency problem (Omotosho and Anyigba, 2019); and innovative behavior and firm performance (Dess et al., 1997). Therefore, the authors discovered from the past literature that the influencing link of EStrg between entrepreneurs' social capital (trust) and entrepreneurs' startup behavior is to be further studied.

It is understood that for firms, EStrg plays a key role in influencing individual behavior. Although there are avoidable known situations where entrepreneurial strategy is convinced not to be taken such as government policies, environmental rules, cultural and social standards; but myriad studies testified that EStrg treated it as an innovative tool to validate trustable entrepreneurial process that increases positive entrepreneurs' behavior (Arora and Nandkumar, 2011). However, entrepreneurs' startup behavior can be influenced by EStrg and independent of social capital (trust) (Sperber and Linder, 2019). Therefore, EStrgy influenced further on entrepreneurs' startup behavior by the interaction through social capital (Khan et al., 2019). In the present research, the authors emphasized on entrepreneurial strategy as a strategic process that function as a moderator between trust (trust on experts, media & Government) and entrepreneurs' startup behavior.

Entrepreneurs that are using entrepreneurial strategy considered their firms as competitive enough: to influence entrepreneurial behaviors and firms performance in fruitful ways; risk taking uses proactiveness for change, innovation, and best utilization of resources by learning from past mistakes (Russell and Russell, 1992; Marom et al., 2019). These entrepreneurial firms further believe that they are competitive and are able to use social capital (trust) as a multi-criterion for positive utilization of startup behavior. Hence, the authors argued that although social capital (trust) can influence entrepreneurs to initiate startup behaviors for new ventures, but entrepreneurs are also required to feel positive and situationally encouraged thorough entrepreneurial strategy for positive startup behavior. Therefore, this study suggested that entrepreneurs' social capital (trust on media, government, experts & enterprises) encourage entrepreneurs to use entrepreneurial strategy at low and medium levels. It may lead to a positive startup behavior; in contrast to those entrepreneurs that interact through high entrepreneurial strategy, do not believe in trust and fail to show any positive startup behavior by interacting through social capital. Hence, on these arguments for low and high entrepreneurial strategy, the study proposes that:

***H5(a):*** *Low entrepreneurial strategy highly moderate between trust on expert & enterprises and startup behaviors as compared to high entrepreneurial strategy*.
***H5(b):*** *Low entrepreneurial strategy highly moderate between trust on media and startup behaviors as compared to high entrepreneurial strategy*.
***H5(c):*** *Low entrepreneurial strategy highly moderate between trust on Government and startup behaviors as compared to high entrepreneurial strategy*

### Moderated mediation

Entrepreneurial strategy moderates as the link between proactive orientation and entrepreneurial performance (Gao et al., 2018). In other studies, entrepreneurial strategy is moderating the positive impact between contingent environment and agency problem (Omotosho and Anyigba, 2019). These scholars proposed to analyze moderated mediation impact of entrepreneurial strategy. The authors could not find literature on moderated mediation role of entrepreneurial strategy for individual behavior and firm success. Thus, to fulfill this gap, the authors hypothesized as:

***H6(a):*** *Low entrepreneurial strategy highly moderates the indirect mediation impact of startup behaviors between trust on experts & enterprises and entrepreneurial success (individual success and firm success) as compared to high entrepreneurial strategy*.
***H6(b):*** *Low entrepreneurial strategy highly moderates the indirect mediation impact of startup behaviors between trust on media and entrepreneurial success (individual success and firm success) as compared to high entrepreneurial strategy*.
***H6(c):*** *Low entrepreneurial strategy moderates the indirect mediation impact of startup behaviors between trust on government and entrepreneurial success (individual success and firm success) as compared to high entrepreneurial strategy*.

## Research methods

### Population, sample, and data collection

**Figure 1:**
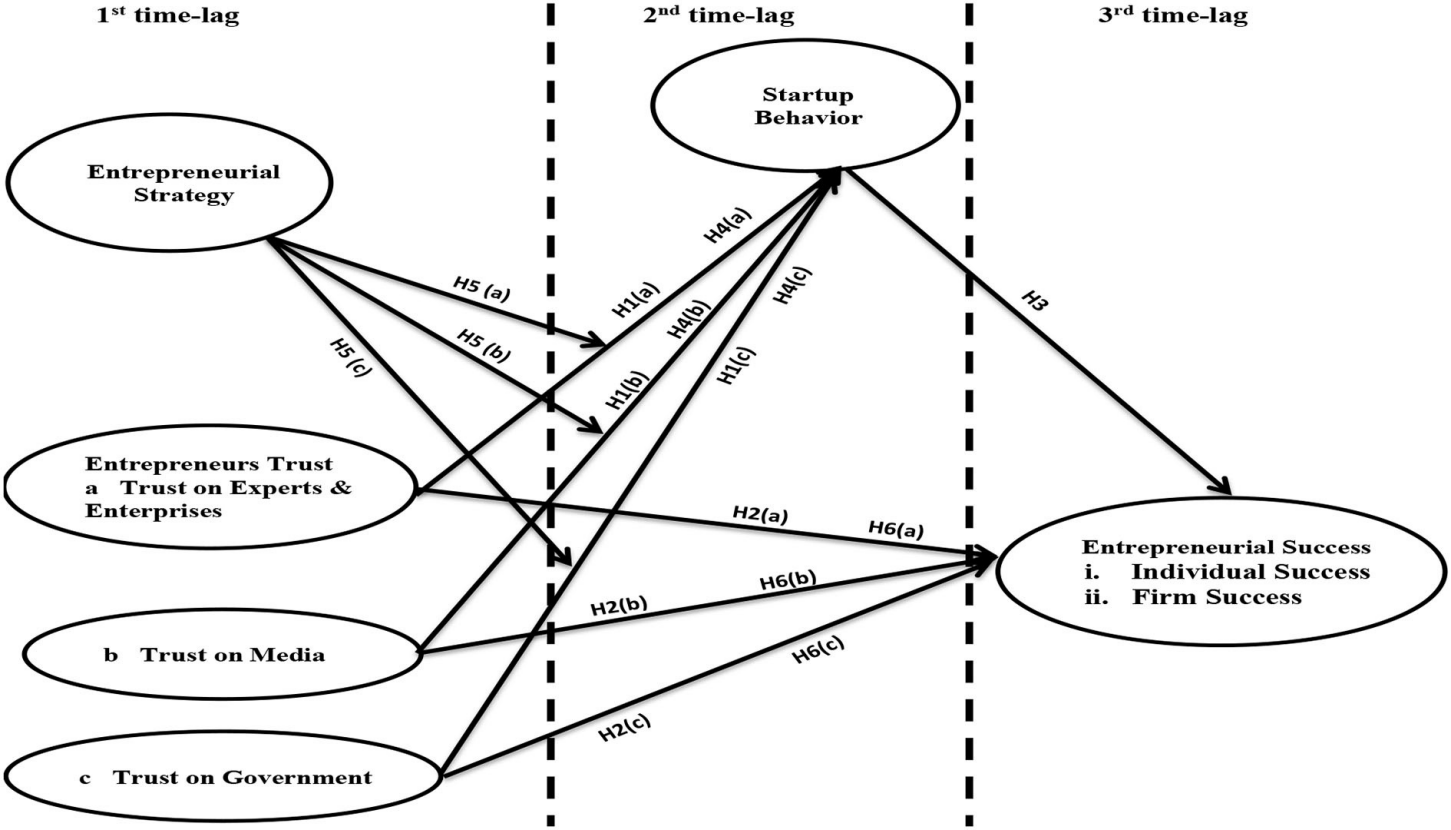
Conceptual model. H4a, H4b, and H4c showing mediating impact; H6a, H6b, and H6c showing moderated mediation impact.

The authors secured a list of 6,500 “*industrial firms”* working as “*manufacturing concerns”* in Pakistan. Their emails were obtained from the websites of recognized Chambers of Commerce. A simple random sampling was applied for the selection of 650 respondents i.e., every 10th firm from the list of 6,500 firms. This research used a survey method and received 540 responses from industrial owners, top-level executives/employees, and key decision makers. This research used time-lagged method (time lag of 2 weeks) for data collection at three different stages (see Figure 1). Data for the independent variable (entrepreneurs trust on experts & enterprises, media, and government) and moderating variable (entrepreneurial strategy) were collected at the first time-lag. Data for mediating variable (startup behavior) were collected at the second time-lag, whereas data for the dependent variable (entrepreneurial individual success and firm success) were collected at the end. Time-lag method is one of the old data collection techniques, which are used to control common method biasness (McArdle and Woodcock, 1997). The variable used for this study includes social capital (i.e., trust) and individual entrepreneurial success for which respondents may mark the questionnaire according to their social desire; hence, the authors decided time-lag technique as a methodological treatment to control social desirability response (Ganster et al., 1983). A mismatched 35 responses at the three time-lags were deleted. Data normality and missing values were found in order. This research used 505 valid responses for analysis.

### Construct measurement

The survey questionnaire covered questions related to the demographics of the respondents as well as questions for the variables used in conceptual model. We used 7-point Likert scale.

#### Entrepreneurs trust on experts & enterprises

A 10-item scale of entrepreneurs trust on experts & enterprises as adapted from Rehman et al. (2020). Example items include “I trust that our experts and enterprises have the ability to make a correct evaluation of entrepreneurial projects”; and “I trust that our experts and enterprises have the ability to gauge the real potential of investments made under entrepreneurial projects.”

#### Entrepreneurs trust on media

A 6-item scale of entrepreneurs trust on media as adapted from Rehman et al. (2020). Example items include “I trust that media reports on entrepreneurial projects are reliable source”; and “I trust that media reports on entrepreneurial projects are timely available.”

#### Entrepreneurs trust on government

A 6-item scale of entrepreneurs' trust on government as adapted from Rehman et al. (2020). Example items include “I trust that decisions made by Government for entrepreneurial projects are essential”; and “I trust that decisions made by Government for entrepreneurial projects are timely.”

#### Startup behavior

A 6-item scale of startup behavior as adapted from Zanger and Geissler (2018) and Harding et al. (2002). Example item included “I talked with others to seek an idea for new startup,” and “I am spending time in searching new startup.”

#### Entrepreneurial strategy

A 5-item scale for entrepreneurial strategy as adopted from Williams and Lee (2011). Example item included “our firm has developed a strategy that encourages high levels of risk-taking” and “our firm has developed a strategy that encourages high levels of innovation.”

#### Entrepreneurial success

A 4-item scale for entrepreneurial individual success and a 2-item scale for entrepreneurial firm success as adopted from Fatoki (2018). Sample items included “I think that my business is growing,” and “growth in firm sales.”

## Results

### Data analysis

**Figure 2:**
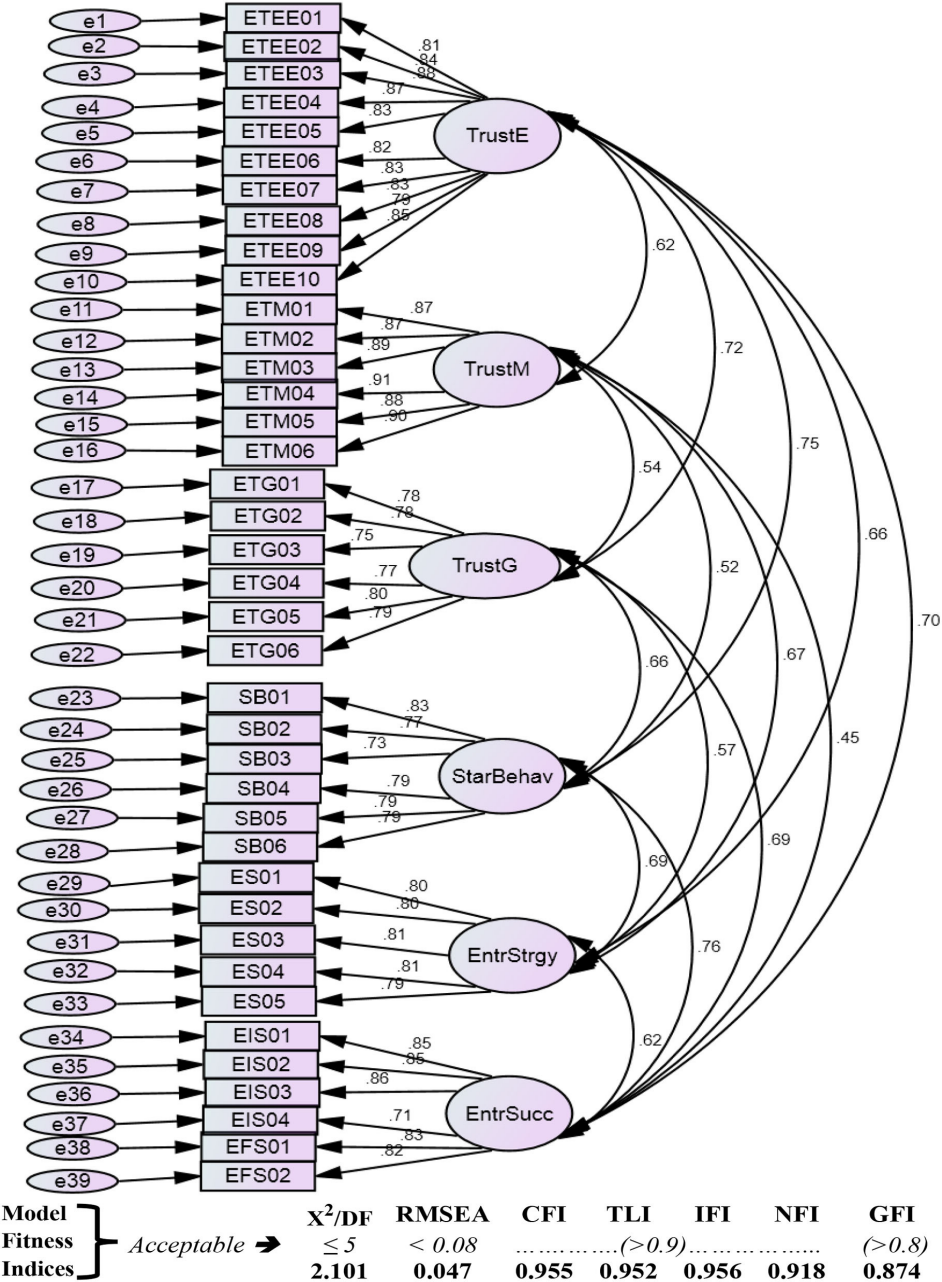
Model fitness.

To analyze the hypothesis for the six studied variables, the authors run structural equation modeling (SEM) in Amos and SPSS. Amos is helpful to confirm model fitness, whereas SPSS in general and PROCESS technique in specific are considered as a reliable technique to comply with regression assumptions (Byrne, 2010). PROCESS technique further helped the authors to confirm the robustness of the data and results for hypothesis testing. PROCESS deals with the questionnaire data for the observed variables through its individual items and providing results for latent variables (Hayes, 2009, 2013). In the conceptual model, the entrepreneurs' situational factor (i.e., entrepreneurial strategy) is explicitly formative due to its high and low effect on the entrepreneurs' dispositional factor (i.e., startup behavior), so applying SEM in Amos for model fitness and hypothesis testing in SPSS (PROCESS MACRO) are suitable statistical techniques to analysis hypothesis (Pallant, 2013). PROCESS model 4 was used for direct path as well as mediation analysis, whereas model 7 was used for moderation as well as mediated mediation. Confirmatory factor analysis was run to analyze model fitness indices (see Figure 2).

#### Factor loading

The authors analyzed factors loading of 39 observed factors consisted of 6 unobserved variables. Factor loadings technique is used to determine the variability within the factors, relationship with the observed variable, and tendency for explaining the observed variable by an individual factor (Shevlin and Miles, 1998). The values of each factor loading were found >0.3 (see Table 1).

**Table 1:** Factor loadings.

**Items**	**Statement**	**Factor loadings**
	**Entrepreneurs trust on experts & enterprises** (α = 0.856) I trust that our experts and enterprises…	
ETEE01	…have the ability to make correct evaluation of entrepreneurial projects	0.861
ETEE02	…have the ability to gauge real potential of investments made under entrepreneurial projects	0.965
ETEE03	…have expressed their opinion on entrepreneurial projects	0.868
ETEE04	…provided a way forward of entrepreneurial projects	0.864
ETEE05	…have performed their responsibilities for entrepreneurial projects	0.754
ETEE06	…are working under legal frameworks	0.807
ETEE07	…are accountable for their works	0.842
ETEE08	…are supportive for business	0.721
ETEE09	…are equipped with required technology	0.747
ETEE10	…are cost effective	0.804
	**Entrepreneurs trust on media** (α = 0.892) I trust that media reports on entrepreneurial projects are…	
ETM01	… reliable source	0.855
ETM02	… timely available	0.885
ETM03	… accurate	0.906
ETM04	… understandable	0.885
ETM05	… not biased	0.911
ETM06	… helpful for awareness	0.929
	**Entrepreneurs trust on Government** (α = 0.872) I trust that decisions made by Government for entrepreneurial projects….	
ETG01	…are essential	0.697
ETG02	…are timely	0.813
ETG03	…are in best interest of the Country	0.769
ETG04	…would strengthen relationship with neighboring countries	0.845
ETG05	…integrate multiple achievable aspects	0.864
ETG06	…by considering the effectiveness of the Projects	0.720
	**Startup behavior** (α = 0.865)	
SB01	I am putting efforts for early-stage entrepreneurial activities for new startup	0.785
SB02	I talked with others to seek an idea for new startup	0.901
SB03	I am spending time in searching new startup	0.837
SB04	I am thinking to invest money as next 12 months will be better for new startup	0.803
SB05	I will prefer to invest for new startup instead to invest elsewhere	0.604
SB06	I found someone who will start a new business in next 12 months	0.694
	**Entrepreneurial strategy** (α = 0.867) Our firm…	
ES01	…has developed a strategy that encourages high levels of risk-taking	0.733
ES02	…has developed a strategy that encourages high levels of innovation	0.816
ES03	…has developed a strategy that encourages high levels of proactivity	0.742
ES04	…is looking for new ways to combine existing resources in pursuit of innovative products	0.824
ES05	…is able to learn from past mistakes	0.803
	**Entrepreneurial success (entrepreneurial individual success and firm success)** (α = 0.870)	
EIS01	I am successful as I am personally satisfied with my life and business	0.780
EIS02	I do only that which I want to do in life and business	0.905
EIS03	I think that my business is growing	0.872
EIS04	I achieve the business goals I set out to achieve	0.791
EFS01	Growth in firm sales	0.888
EFS02	Growth in firm profits	0.750

#### Data normality

After examining factor loading, we analyzed data normality in terms of its mean, SD (near to 1), skewness (values within ±2 are acceptable), and kurtosis (values within ±3 are acceptable) (Curran et al., 1996). Values for Mean, standard deviation (SD), reliability, validity, and correlations between the studied six variables are given in Table 2. The demographic statistics for studied six variables, i.e., trust on experts & enterprises (TEE; *M* = 5.982*, SD* = 0.980), trust on media (TM; *M* = 5.429, *SD* = 0.1.306*)*, trust on government (TG; *M* = 5.6.084*, SD* = 0.0.905), startup behavior (StBeh; *M* = 5.959*, SD* = 0.872), entrepreneurial strategy (EStrg; *M* = 5.810*, SD* = 1.016), and entrepreneurial success (ESuc; *M* = 5.810, *SD* = 1.016).

**Table 2:** Correlation, reliability and validity, mean and standard deviation.

	**Mean**	**SD**	**CR**	**ICR**	**AVE**	**TEE**	**TM**	**TG**	**StBeh**	**EStrg**	**ESuc**
**TEE**	5.982	0.980	0.958	0.856	0.697	**(0.835)**					
**TM**	5.429	1.306	0.955	0.892	0.787	0.591	**(0.887)**				
**TG**	6.084	0.905	0.903	0.872	0.608	0.669	0.506	**(0.779)**			
**StBeh**	5.959	0.872	0.905	0.865	0.613	0.706	0.490	0.596	**(0.783)**		
**EStrg**	5.810	1.016	0.902	0.867	0.648	0.610	0.627	0.513	0.625	**(0.821)**	
**ESuc**	6.151	0.875	0.925	0.870	0.673	0.667	0.430	0.630	0.700	0.571	**(0.805)**

Correlation coefficients are significant.

Values of sqrt-AVE are showing in diagonal.

TEE, Trust on Experts & Enterprises; TM, Trust on Media; TG, Trust on Government; StBeh, Startup Behavior; EStrg, Entrepreneurial Strategy; ESuc, Entrepreneurial Success.

#### Correlation

The link between six studied variables was also examined by correlation test (see Table 2). Results show that all correlations are significant at *p* < 0.001. Trust on experts & enterprises is correlated with trust on media (TM; *r* = 0.591), trust on Government (TG; *r* = 0.669), startup behavior (StBeh; *r* = 0.706), entrepreneurial strategy (EStrg; *r* = 0.610), and ESuc (*r* = 0.667). TM is correlation with TG (*r* = 0.506), StBeh (*r* = 0.490), EStrg (*r* = 0.627), and ESuc (*r* = 0.430). TG is correlated with StBeh (*r* = 0.596), EStrg (*r* = 0.513), and ESuc (*r* = 0.630). StBeh is correlated with EStrg (*r* = 0.625) and ESuc (*r* = 0.700). EStrg is correlated with ESuc (*r* = 0.571).

After the correlation test, the authors also examined validity and reliability statistics (see Table 2). The values for AVE are >0.5 and confirmed the convergent validity (Lowry and Gaskin, 2014). The values of Sqrt-AVE were found > corresponding correlation values and confirmed discriminant validity (Tabachnick et al., 2007). The reliability for the six variables was verified through composite reliability along with Cronbach alpha (acceptable > 0.5) (Fornell and Larcker, 1981).

#### Respondents profile

Data were received all-round Pakistan. Male data providers are 85% due to Pakistani patriarchy society. Between 78% and 80% of respondents are having master's degree, 31–50 years of age, and experience above 6 years (see Table 3).

**Table 3:** Demographic profile.

		**Frequency**	**Percentage**
**Gender**	Male	431	85.3
	Female	74	14.7
**Age**	30 & below	68	13.5
	31–40	240	47.5
	41–50	160	31.7
	50 and above	37	7.5
**Education**	Bachelors (14 years & below)	105	20.8
	Masters (16 years & above)	400	79.2
**Experience**	5 & below	57	11.3
	6–10	113	22.4
	11–15	133	26.3
	16–20	149	29.5
	21 & above	53	10.5
**Province**	Islamabad	86	17.0
	Punjab	242	47.9
	Sindh	78	15.4
	Baluchistan	40	7.9
	KPK	24	4.8
	AJK	25	5.0
	Gilgit	10	2.0

#### Direct path regressions/hypothesis testing

The result for direct nexus depicts that the impact of trust on experts & enterprises (TEE) has significant impact on startup behavior (StBeh; *b* = 0.628; *p* < 0.001), trust on media (TM) has significant impact on startup StBeh (*b* = 0.327; *p* < 0.001), trust on government (TG) has significant impact on startup StBeh (*b* = 0.574; *p* < 0.001). Hence, hypotheses 01(a), 1(b), & 1(c) are accepted. Similarly, the impact of TEE has significant impact on entrepreneurial success (ESuc; *b* = 0.308; *p* < 0.001), TM has significant impact on ESuc (*b* = 0.077; *p* < 0.01), TG has significant impact on ESuc (*b* = 0.319; *p* < 0.001). Hence, hypotheses 02(a), 2(b), & 2(c) are accepted. The direct impact of StBeh on ESuc has also a significant impact (*b* = 0.646; *p* < 0.001). Hence, hypothesis 3 is accepted (see Table 4).

**Table 4:** Results of structural model to test the hypotheses.

**Path correlation**	***B***	**SE**	***T***	***p*-Value**	**LLCL**	**ULCL**	**Hyp**.	**Result**
**TEE → SB**	0.628	0.028	22.36	***	**—**	**—**	**H1(a)**	**Accepted**
**TM → SB**	0.327	0.024	12.62	***	**—**	**—**	**H1(b)**	**Accepted**
**TG → SB**	0.574	0.034	16.66	***	**—**	**—**	**H1(c)**	**Accepted**
**TEE → ESuc**	0.308	0.038	8.15	***	**—**	**—**	**H2(a)**	**Accepted**
**TM → ESuc**	0.077	0.024	3.16	**	**—**	**—**	**H2(b)**	**Accepted**
**TG → ESuc**	0.319	0.036	8.95	***	**—**	**—**	**H2(c)**	**Accepted**
**SB → ESuc**	0.646	0.036	17.76	***	**—**	**—**	**H3**	**Accepted**
**TEE → SB → ESuc**	0.288	0.050	**—**	**—**	0.189	0.384	**H4(a)**	**Accepted**
**TM → SB → ESuc**	0.211	0.034	**—**	**—**	0.147	0.280	**H4(b)**	**Accepted**
**TG → SB → ESuc**	0.290	0.044	**—**	**—**	0.202	0.376	**H4(c)**	**Accepted**

TEE, trust on experts & enterprises; TM, trust on media; TG, trust on government; SB, startup behavior; estrg, entrepreneurial strategy; esuc, entrepreneurial success.

**Significant level p < 0.005.

***Significant level p < 0.001.

#### Indirect path for mediation impact

The mediation impact of TEE through StBeh has a significant and positive impact on ESuc (*b* = 0.288; CI = LLCL 0.189, ULCL 0.384), the mediation of EM through StBeh has a significant and positive impact on ESuc (*b* = 0.211; CI = LLCL 0.147, ULCL 0.280). The mediation of EG through StBeh has a significant and positive impact on ESuc (*b* = 0.290; CI = LLCL 0.202, ULCL 0.376) and hypothesis 4(a), 4(b), & 4(c) are accepted (see Table 4).

#### Moderated impact at low, medium, & high values of entrepreneurial strategy

**Figure 3:**
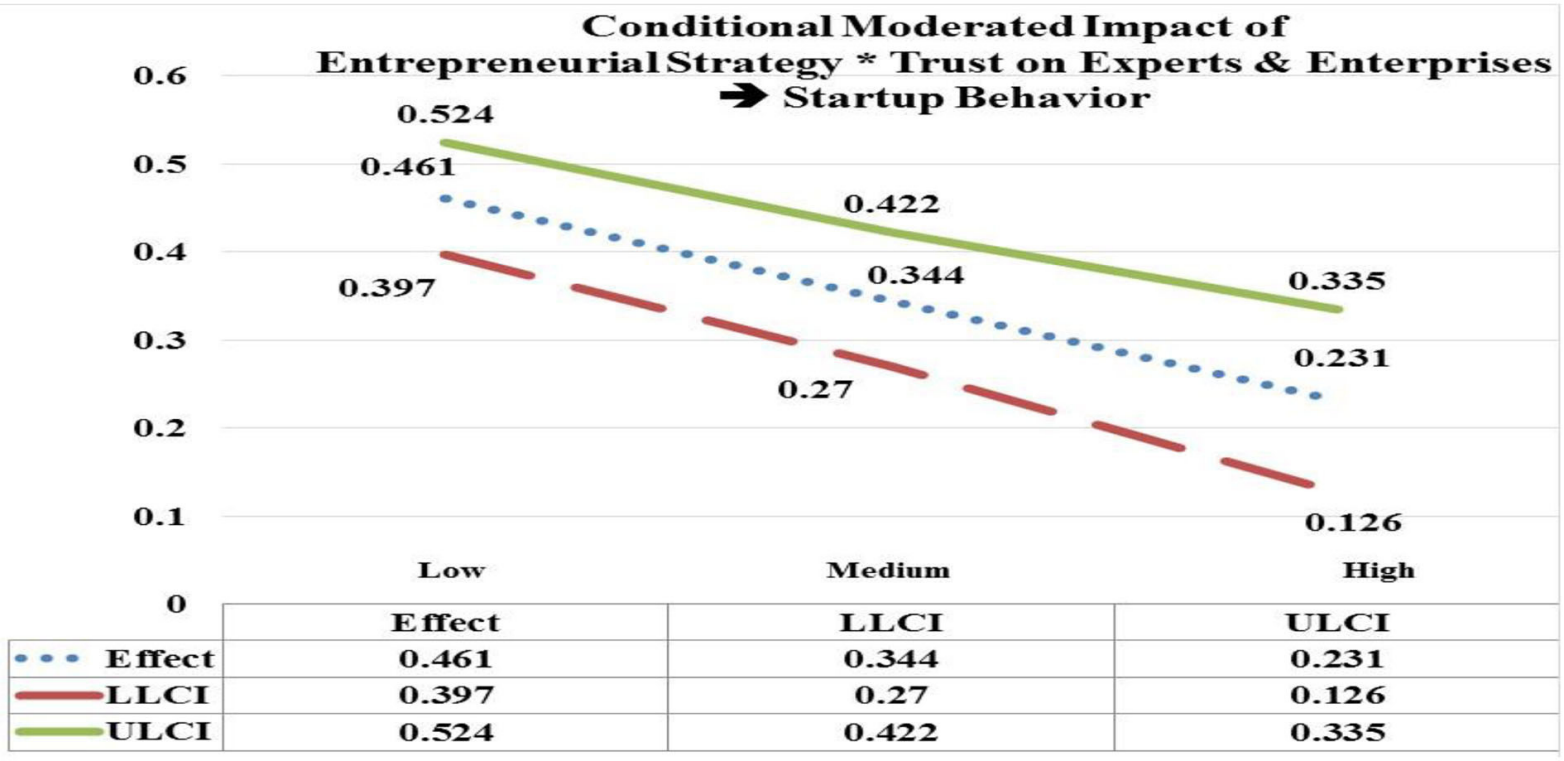
Moderated impact of entrepreneurial strategy * trust on experts & enterprises → startup behavior.

The moderated impact of entrepreneurial strategy (EStrg) at low, medium, and high values by interaction with trust on experts & enterprises (TEE) has significant and positive impact on startup behavior (StBeh) (low EStrg = 4.7938, *b* = 0.461; CI = LLCL 0.397, ULCL 0.524); (medium EStrg = 5.8103, *b* = 0.344; CI = LLCL 0.270, ULCL 0.422); (high EStrg = 6.8268, *b* = 0.053; CI = LLCL 0.126, ULCL 0.335). Hypothesis 5(a) is accepted as the significant impact of EStrg^*^TEE is moderated more on StBeh at low values (e.g., *b* = 0.461) in contrast with its moderated impact (e.g., *b* = 0.053) at high values (see Figure 3).

**Figure 4:**
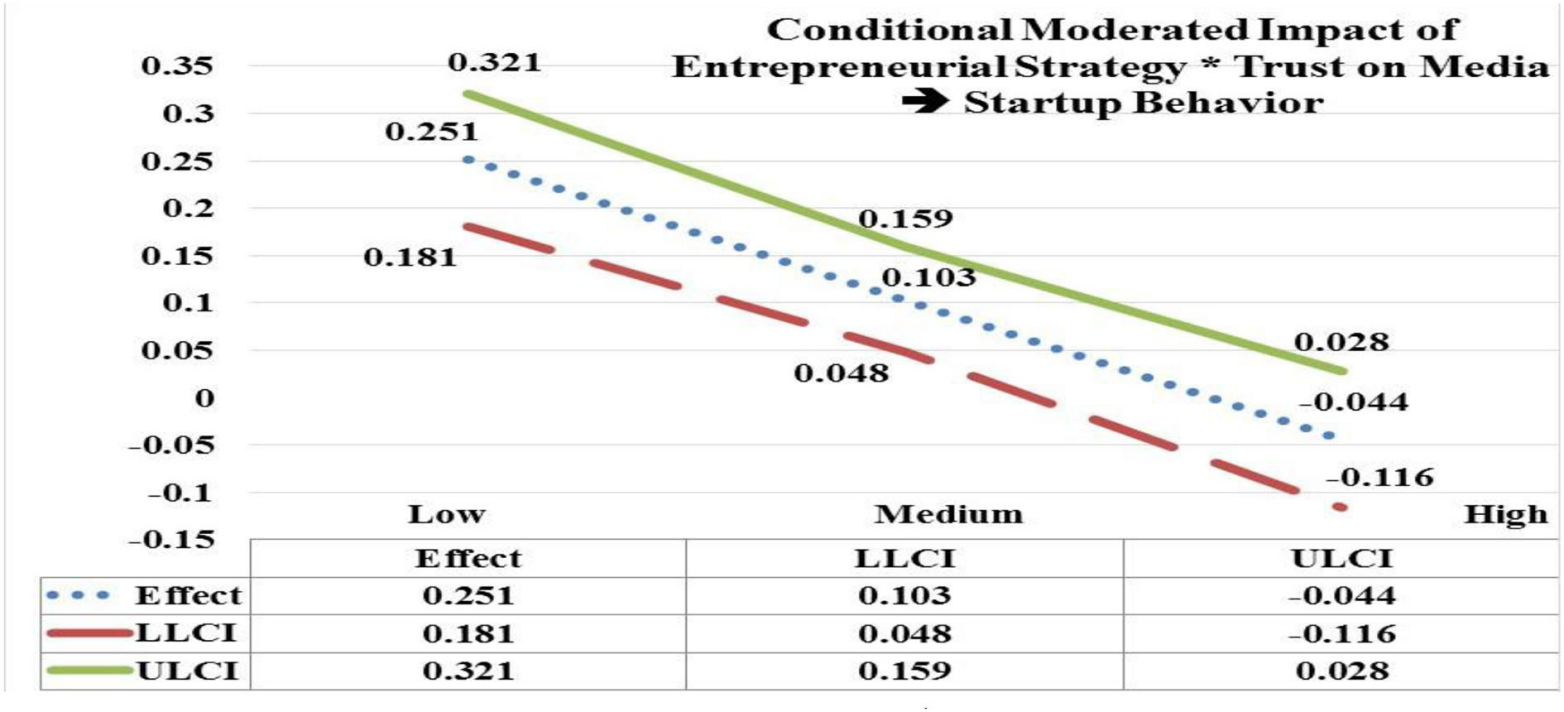
Moderated impact of entrepreneurial strategy * trust on media → startup behavior.

The moderated impact of EStrg at low and medium values by interaction with trust on media (TM) has significant and positive impact on startup behavior (StBeh) (low EStrg = 4.7938, *b* = 0.251; CI = LLCL 0.181, ULCL 0.321); (medium EStrg = 5.8103, *b* = 0.103; CI = LLCL 0.048, ULCL 0.159). In contrast, moderated impact of EStrg at high values by interaction with TM is insignificant on StBeh (high EStrg = 6.8268, *b* = **–**0.044; CI = LLCL **–**0.116, ULCL 0.028). Hypothesis 5(b) is accepted as the significant impact of EStrg^*^TM is moderated more on StBeh at low values (e.g., *b* = 0.251) in contrast with its insignificant moderated impact at high values (see Figure 4).

**Figure 5:**
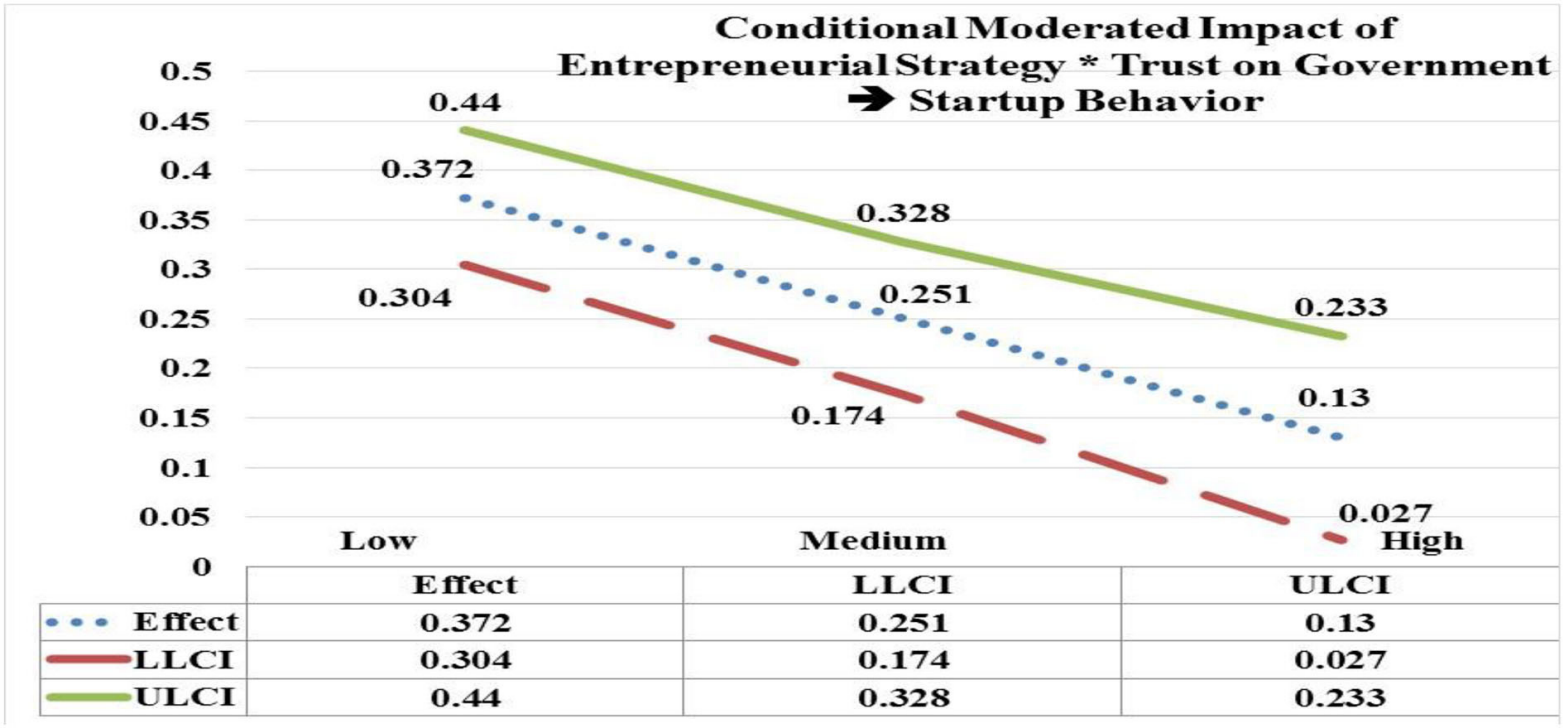
Moderated impact of entrepreneurial strategy * trust on government → startup behavior.

The moderated impact of EStrg at low, medium, and high values by interaction with trust on government (TG) is significant and positive on startup behavior (StBeh) (low EStrg = 4.7938, *b* = 0.372; CI = LLCL 0.304, ULCL 0.440); (medium EStrg = 5.8103, *b* = 0.251; CI = LLCL 0.174, ULCL 0.328); (high EStrg = 6.8268, *b* = 0.130; CI = LLCL 0.027, ULCL 0.233). Hypothesis 5(c) is accepted as the significant impact of EStrg^*^TG is moderated more on StBeh at low values (e.g., *b* = 0.371) in contrast with its moderated impact (e.g., 0.130) at high values (see Figure 5).

#### Moderated mediation impact at low, medium, and high values of entrepreneurial strategy

**Figure 6:**
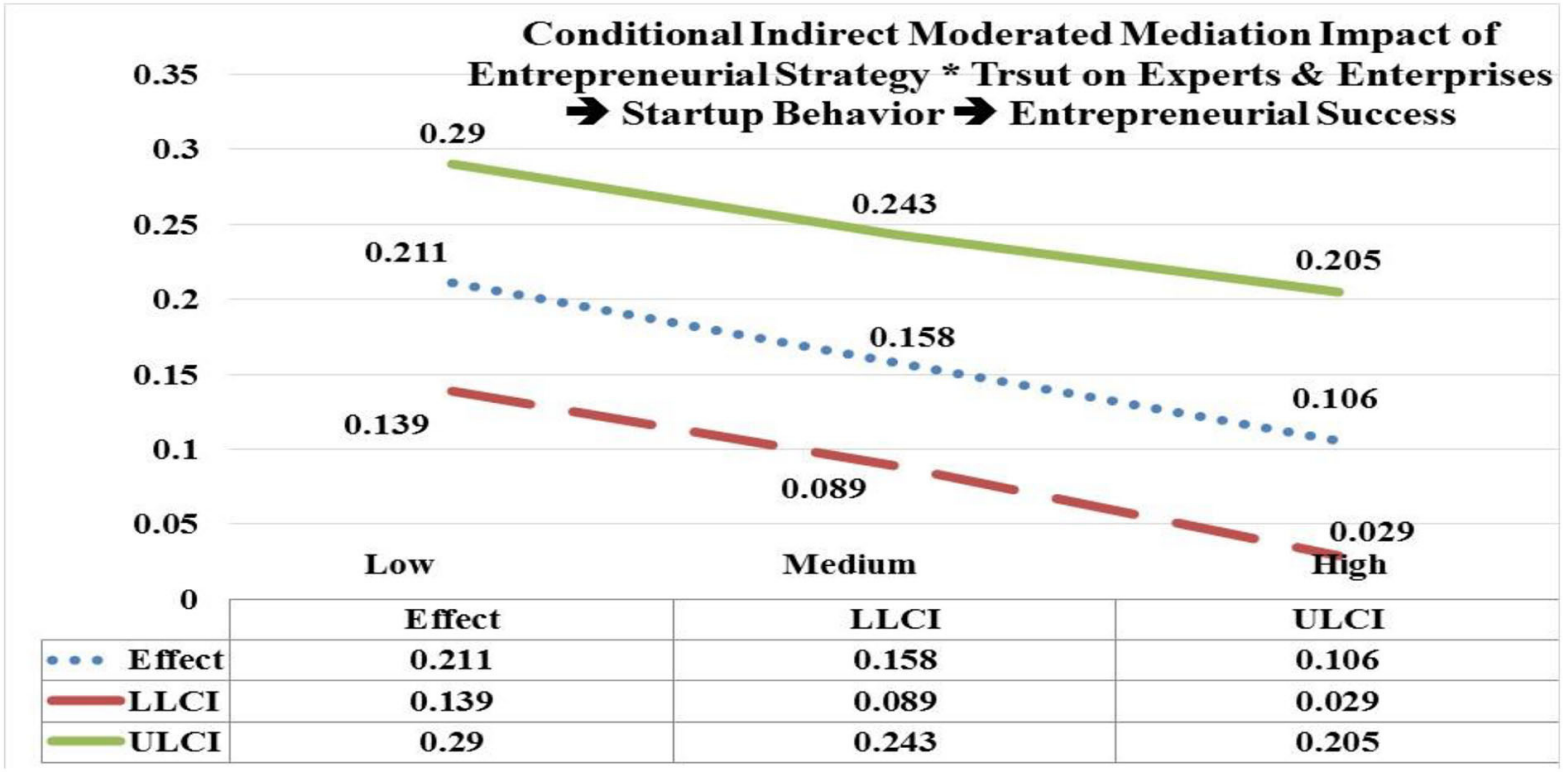
Moderated mediation impact of entrepreneurial strategy * trust on experts & enterprises → startup behavior → entrepreneurial success.

The moderated mediation of entrepreneurial strategy (EStrg) at low, medium, and high values by interaction with trust on experts & enterprises (TEE) through startup behavior (StBeh) has significant and positive impact on entrepreneurial success (ESuc) (low EStrg = 4.7938, *b* = 0.211; CI = LLCL 0.139, ULCL 0.290); (medium EStrg = 5.8103, *b* = 0.158; CI = LLCL 0.089, ULCL 0.243); (high EStrg = 6.8268, *b* = 0.106; CI = LLCL 0.029, ULCL 0.205). Hypothesis 6(a) is accepted as the significant impact of EStrg^*^TEE is moderated mediating through StBeh more on ESuc at low values (e.g., *b* = 0.211) in contrast with its impact (e.g., *b* = 0.106) at high values (see Figure 6).

**Figure 7:**
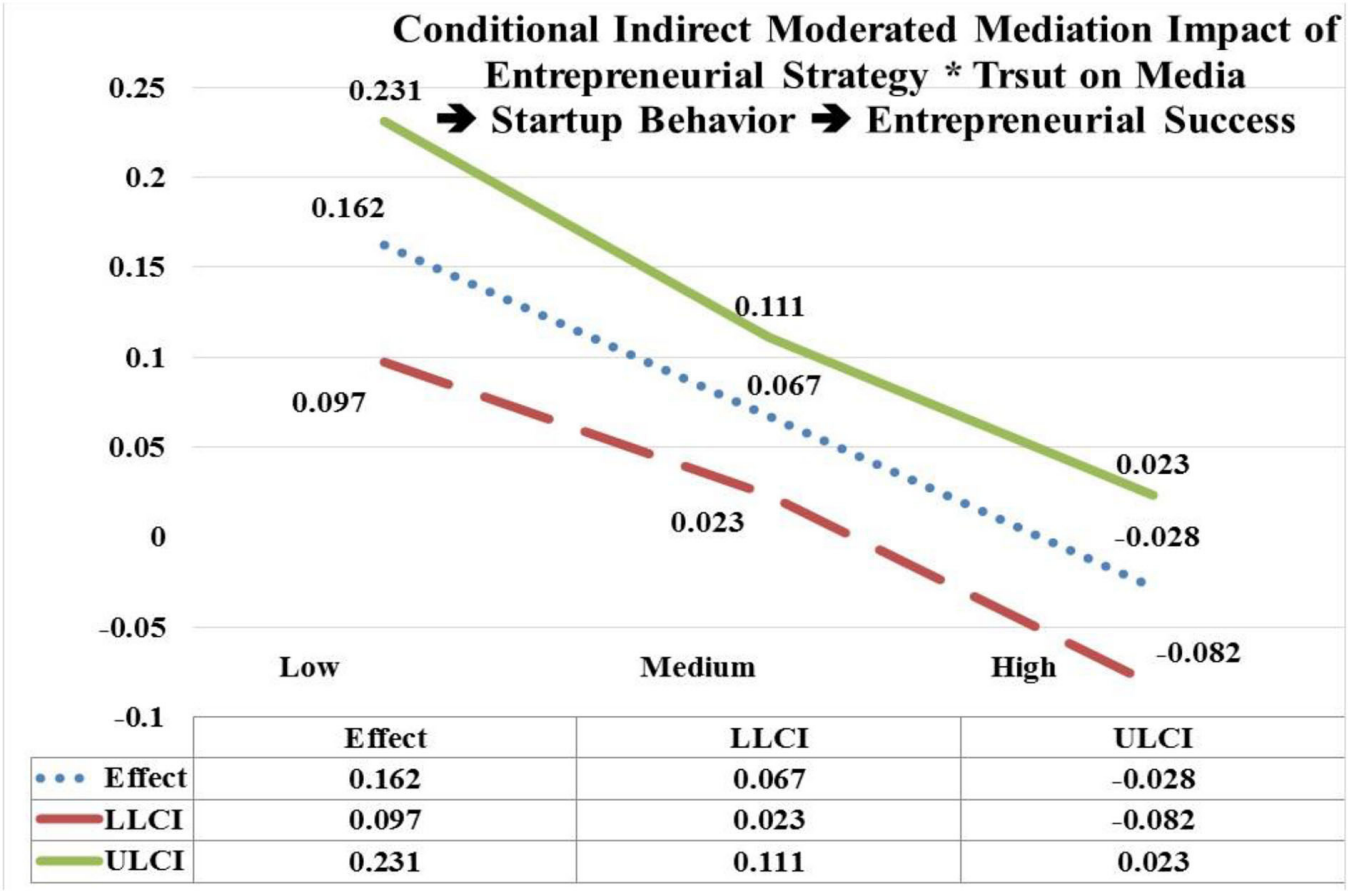
Moderated mediation impact of entrepreneurial strategy * trust on media → startup behavior → entrepreneurial success.

The moderated mediation of entrepreneurial strategy (EStrg) at low, and medium values by interaction with trust on media (TM) through startup behavior (StBeh) has significant and positive impact on entrepreneurial success (ESuc) (low EStrg = 4.7938, *b* = 0.162; CI = LLCL 0.097, ULCL 0.231); (medium EStrg = 5.8103, *b* = 0.067; CI = LLCL 0.023, ULCL 0.111). In contrast, moderated mediating impact of EStrg at high values by interaction with TM through StBeh is insignificant on ESuc (high EStrg = 6.8268, *b* = −0.028; CI = LLCL −0.082, ULCL 0.023). Hypothesis 6(b) is accepted as the significant impact of EStrg^*^TM is moderated mediating through StBeh more on ESuc at low values (e.g., *b* = 0.162) in contrast with its insignificant impact at high values (see Figure 7).

**Figure 8:**
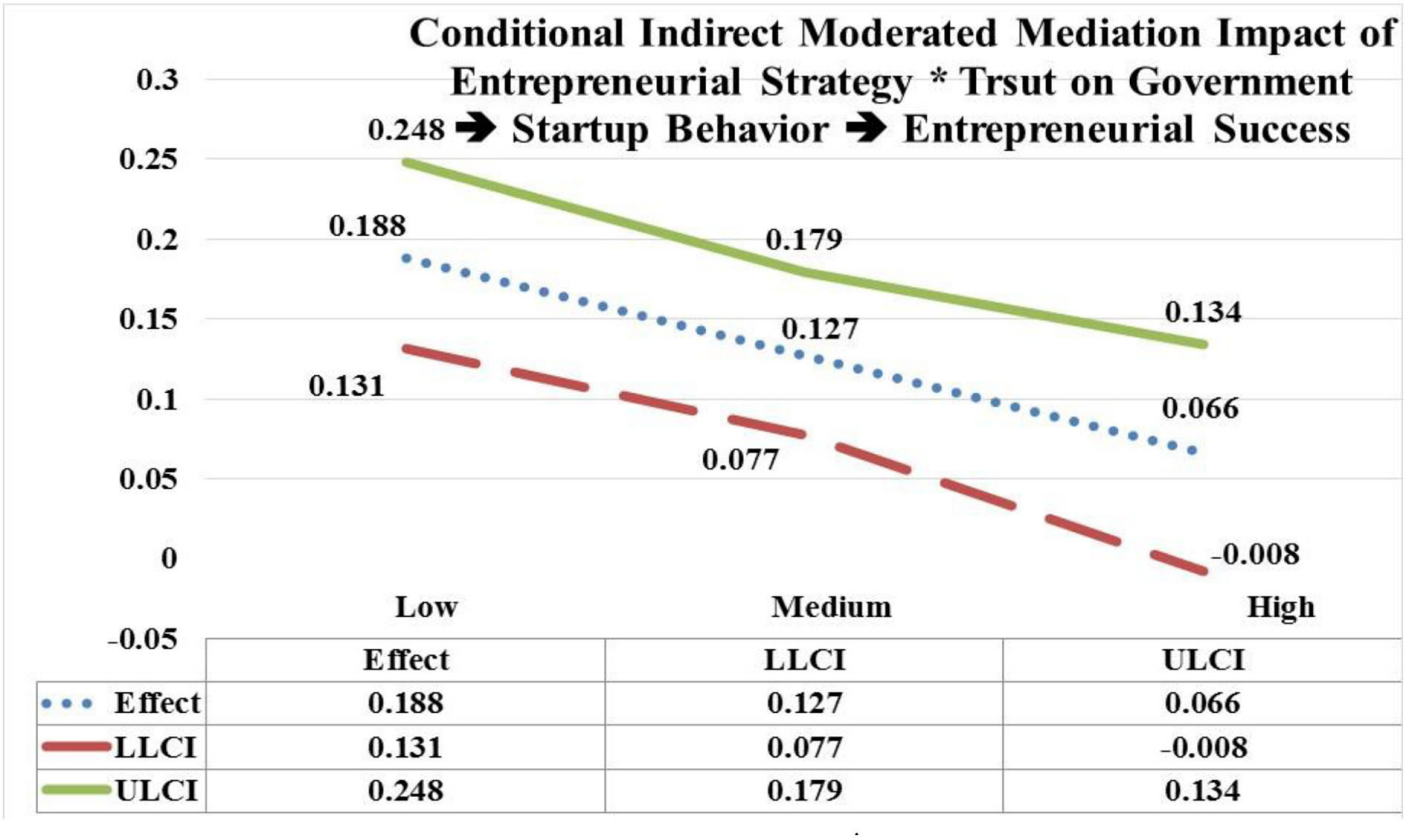
Moderated mediation impact of entrepreneurial strategy * trust on government → startup behavior → entrepreneurial success.

The moderated mediation of entrepreneurial strategy (EStrg) at low, and medium values by interaction with trust on government (TG) through startup behavior (StBeh) has significant and positive impact on entrepreneurial success (ESuc) (low EStrg = 4.7938, *b* = 0.188; CI = LLCL 0.131, ULCL 0.248); (medium EStrg = 5.8103, *b* = 0.127; CI = LLCL 0.077, ULCL 0.179). In contrast, moderated mediating impact of EStrg at high values by interaction with TG through StBeh has an insignificant impact on ESuc (high EStrg = 6.8268, *b* = −0.066; CI = LLCL −0.008, ULCL 0.134). Hypothesis 6(c) is accepted as the impact of EStrg^*^TG is moderated mediating through StBeh more on ESuc at low values (e.g., *b* = 0.188) in contrast with its insignificant impact at high values (see Figure 8).

## Discussion

Notwithstanding the voluminous studies on social capital and various outcomes of entrepreneurship, the stock of this study has shown significance on these two constructs by creating connections for possible nexus between them. To some extent, the study in hand creates a possible connection between them by examining the relationship between social capital (trust) and entrepreneurial success (individual and firm success). It further links trust with success by creating entrepreneurial dispositional factor (startup behavior) as the underlying mechanism (mediator) and entrepreneurs' situational factor (entrepreneurial strategy) as a boundary condition (moderator).

In our findings, a direct nexus of trust on three elements of ecosystem (trust on experts & enterprises, trust on media, and trust on government) with startup behavior and entrepreneurial success is found positively significant. Similarly, trust on three elements of ecosystem has positively influenced entrepreneurial success under a mechanism of startup behavior. Among three elements of ecosystem, two elements trust on experts & enterprise and trust on government (under direct and indirect relationship) are influencing startup behavior and entrepreneurial success comparatively more in contrast with trust on media.

Similarly, the entrepreneurs who are highly associated with entrepreneurial strategy need a high resource from trust on experts & enterprise and trust on government for a moderated nexus with startup behavior. However, entrepreneurs have high association with entrepreneurial strategy, when interacting through trust on media, its moderated nexus with startup behavior is found insignificant. Indeed, entrepreneurs having low association with entrepreneurial strategy require less resource from trust for moderated nexus with startup behavior.

Similarly, the entrepreneurs who are highly associated with entrepreneurial strategy need a high resource from trust on experts & enterprises for a moderated mediating nexus through startup behavior with entrepreneurial success. However, entrepreneurs having high association with entrepreneurial strategy when interacting through trust on media and trust on government, its moderated mediating nexus through startup behavior with entrepreneurial success is found insignificant. Precisely, the entrepreneurs having low association with entrepreneurial strategy require less resource from trust on experts & enterprise, trust on media, and trust on government for moderated mediating nexus through startup behavior with entrepreneurial success.

### Implications of findings

This study contributed various dimensions to the literature of social capital, entrepreneurship, and behavioral sciences besides the owners and top executives of manufacturing industry:

*Firstly*, this study added a more intense conception of trust on three elements of ecosystem (social capital: trust on experts & enterprise, trust on media, and trust on government) and enlightened its role in entrepreneurial success. Our results confirmed that the role of trust on experts & enterprises, media, and government is positively influencing for entrepreneurial outcome. This nexus is also positively significant through an underlying mechanism of startup behavior. Our finding also validated attitude-change theory (Sherif et al., 1965) that trust injected positive startup behavior in the entrepreneurs. In reaction, the entrepreneurs treated behavior as an attitude-change (Lorenz et al., 2021; Tormala and Rucker, 2022) and through this changed-behavior, the entrepreneurs achieved entrepreneurial outcome.
*Secondly*, low and medium entrepreneurial strategy positively moderate between the nexus of trust on three elements of ecosystem and startup behavior in contrast where the impact of high entrepreneurial strategy is insignificant when interacting through trust on media. In comparison with our results, the past research highlighted the complex nature of trust for predicting behavioral change (Appelbaum et al., 2004; Bi et al., 2021). When trust is interacting with high entrepreneurial strategy, the owners of manufacturing industry get more resources from trust on experts & enterprises and trust on government for startup behavior. In contrast, the owners of manufacturing industry treated trust on media as multicriteria (Boender et al., 1989) and so far, not ready for startup behavior through trust on media when it interacts through a high entrepreneurial strategy. Precisely, while interacting through entrepreneurial strategy at high, medium, and low values, different forms of trust are substituting each other for positive change in startup behavior (Kerr and Jermier, 1978; Bi et al., 2021). As more as the owners of manufacturing industry get satisfaction from trust on experts & enterprises and trust on government, the other form of trust (trust on media) is less influencing their startup behavior due to multicriteria decision-making and theory of substitute effect (Byrne, 2010).
*Finally*, entrepreneurial strategy is also analyzed as a substitute (Kerr and Jermier, 1978) for trust in predicting entrepreneurial outcomes. As per our results, low entrepreneurial strategy by interaction through trust on three elements of ecosystem is significant for moderated mediation effect on entrepreneurial success through startup behavior. In contrast, when entrepreneurs interact through a high entrepreneurial strategy, its moderated mediation impact is insignificant through trust on media and trust on government. According to Khan et al. (2019), entrepreneurial strategy is necessary for the industry in which interaction of entrepreneurs is required with a large number of firm stakeholders. According to Solovida and Latan (2017), environmental strategy is helpful for environmental performance through proper environmental management. These researchers did not treat “trust” as a mandatory element for performance outcomes. Similarly, the manufacturing industry is also high stakeholders affected industry (Ploywarin et al., 2018). Hence, the owners of manufacturing industry when stuck through trust on media and trust on government for entrepreneurial success may start interacting through other sources (low and medium entrepreneurial strategy) by substituting trust. The other sources (low and medium entrepreneurial strategy) reasonably provide them with the required source for entrepreneurial success, while some elements of trust failed to provide at high entrepreneurial strategy. Hence, our results are in support as well as in contradiction with previous findings (Tsai and Liao, 2017).

## Conclusion

The present research extended the existing knowledge on post-COVID-19 life on earth, entrepreneurial mindset to achieve entrepreneurial success; and the significant role of social capital (trust on three elements of ecosystem i.e., trust on experts & enterprises, trust on media, and trust on government) as an influencing factor for entrepreneurial outcomes (entrepreneurial success i.e., both individual and firm success) through underlying mechanisms (startup behavior) and a boundary condition (high, medium, and low entrepreneurial strategy). It particularly found that social capital (trust) provides many resources to shape and build startup behavior; trust is also helpful to achieve entrepreneurial success through direct involvement of trust with these entrepreneurial outcomes.

Considering a mechanism (mediation) through startup behavior, the social capital (trust) provides sufficient resources for entrepreneurial success as startup behavior is treated as attitude-change for the effect of trust on success. In addition, low and medium entrepreneurial strategy positively moderates the nexus between trust on experts & enterprises, media, and government with startup behavior. But entrepreneurial strategy (at high values) remained insignificant for the nexus of trust on media with startup behavior. Precisely, industrial entrepreneurs treated trust and entrepreneurial strategy as multicriteria against each other for startup behavior. When industrial entrepreneurs are highly associated with entrepreneurial strategy, they consider trust as multicriteria against high entrepreneurial strategy for startup behavior. But when industrial entrepreneurs are less associated with entrepreneurial strategy, they consider low entrepreneurial strategy as multicriteria against trust for startup behavior. Finally, the role of social capital (trust) is more dependent on entrepreneurial success when interacts through moderated mediation effect of entrepreneurial strategy and startup behavior. Low and medium entrepreneurial strategy positively moderated to mediate the nexus between trust on experts & enterprises, government, and media with entrepreneurial success through startup behavior. But high entrepreneurial strategy remained insignificant by interaction through trust on two elements of ecosystem (trust on media and trust on government) for moderated mediation on entrepreneurial success through startup behavior. Indeed, industrial entrepreneurs treated “*trust”* and “*entrepreneurial strategy”* as a substitute against each other for entrepreneurial success. When industrial entrepreneurs are highly associated with entrepreneurial strategy (high entrepreneurial strategy stuck the entrepreneurs through insignificant results for trust on two elements of ecosystem), entrepreneurs treat low and medium entrepreneurial strategy as a substitute for high entrepreneurial strategy. Similarly, when trust on media and trust on government struck the entrepreneurs, they treat other sources (like low and medium entrepreneurial strategy) as a substitute against trust. The availability of other resources (trust on different elements and low & high entrepreneurial strategy as substitute against each other) reasonably provides entrepreneurs the required resource for entrepreneurial success when the substitute failed to provide the resource. Finally, we concluded that trust leads to behavior and success; behavior leads to success; and low entrepreneurial strategy improves this relationship.

### Limitations and future research direction

In spite of data collection at three time-lags to establish effects between six studied variables, this research has some limitations which may be treated in future research. *Firstly*, the current research used a mechanism of startup behavior and boundary condition of entrepreneurial strategy. We recommended using entrepreneurs' psychological capital as mechanism and entrepreneurial governance or using entrepreneurs' resilience as a boundary condition. *Secondly*, this study used trust on three elements of ecosystem i.e., trust on experts & enterprises, media, and government. We also recommended to apply trust on other elements of ecosystem such as goods providers, customers, NGOs employees, and bankers. Further, we recommended to prepare measurement instrument for these elements of ecosystem. *Thirdly*, we selected manufacturing industry that is almost owned by the private sector in Pakistan; however, we recommended that future research may be conducted on the industry owned by both government and private sectors such as air industry or hospital industry. This practice may produce a compression of results in government-owned firms and privately owned firms. *Fourthly*, this research used startup behavior and entrepreneurial strategy as entrepreneurial mindset to achieve success; we recommended to analyze other entrepreneurial mindsets for success such as fear of failure, value creation, entrepreneurial passion, and tendency/intensity of entrepreneurial success. Finally, we deliberated on manufacturing industry which is highly effected by a large number of stakeholders; hence, we recommend that future research may be conducted on the industry which is effected by a smaller number of stakeholders like SMEs.

## Data availability statement

The data analyzed in this study is subject to the following licenses/restrictions. According to the University Ethical Committee, we cannot share the Data. Requests to access these datasets should be directed at: habibgul544@yahoo.com.

## Ethics statement

Ethical review and approval was not required for the study on human participants in accordance with the local legislation and institutional requirements. Written informed consent from the patients/participants was not required to participate in this study in accordance with the national legislation and the institutional requirements.

## Author contributions

ZR has taken the overall responsibilities of the manuscript and give the idea of the topic. MA has written the introduction part, the literature review, and compiled the discussion part. HG is a senior researcher and he has provided a technical support throughout the manuscript. JR run the statistical analysis, taken the responsibility of data collection, and helped in the methodology part. All authors contributed to the article and approved the submitted version.

## Conflict of interest

The authors declare that the research was conducted in the absence of any commercial or financial relationships that could be construed as a potential conflict of interest.

## Publisher's note

All claims expressed in this article are solely those of the authors and do not necessarily represent those of their affiliated organizations, or those of the publisher, the editors and the reviewers. Any product that may be evaluated in this article, or claim that may be made by its manufacturer, is not guaranteed or endorsed by the publisher.
